# Genomic characterization of *Staphylococcus aureus* isolated from mastitis in small ruminants in Sardinia, Italy

**DOI:** 10.3389/fmicb.2025.1661122

**Published:** 2025-12-02

**Authors:** Amira A. Moawad, Simone Dore, Stefano Lollai, Hosny El-Adawy, Hanka Brangsch, Stefan Monecke, Ralf Ehricht, Sascha D. Braun, Heinrich Neubauer, Herbert Tomaso

**Affiliations:** 1 Friedrich-Loeffler-Institut (Federal Research Institute for Animal Health), Institute of Bacterial Infections and Zoonoses, Jena, Germany; 2 Animal Health Research Institute, Agriculture Research Center (ARC), Giza, Egypt; 3 National Reference Center for Sheep and Goat Mastitis, Experimental Zooprophylactic Institute of Sardinia, Sassari, Italy; 4 Faculty of Veterinary Medicine, Kafrelsheikh University, Kafr El-Sheikh, Egypt; 5 Leibniz Institute of Photonic Technology, Member of the Research Alliance “Leibniz Health Technologies” and the Leibniz Centre for Photonics in Infection Research (LPI), Jena, Germany; 6 InfectoGnostics Research Campus Jena, Center for Applied Research, Jena, Germany; 7 Institute of Physical Chemistry, Friedrich Schiller University Jena, Jena, Germany

**Keywords:** dairy sheep and goats, *Staphylococcus aureus*, clinical mastitis, Sardinia, WGS, MSSA

## Abstract

**Background:**

Staphylococcal mastitis is a common disease of small ruminants causing major economic losses. The problem is particularly significant in the rural areas of the Mediterranean region, where almost two thirds of the global sheep and a quarter of the global goat milk are produced. This study aimed to gain insight into the genotypes, antimicrobial resistance (AMR) profiles and virulence factors of *Staphylococcus aureus* isolated from clinical mastitis in small ruminants’ farms from different 25 dairy herds in six different provinces in Sardinia, Italy between December 2021 and May 2022.

**Methods:**

Thirty- two *S. aureus* were phenotypically identified and confirmed by real-time PCR. Whole-genome sequencing (WGS) was conducted and the sequence data were analyzed to reveal the genetic diversity, AMR markers and virulence genes to draw a conclusion for a current situation of small ruminants’ clinical mastitis in dairy herds in the region and the potential public health risk. Furthermore, the phylogenetic relations between *S. aureus* strains within one farm and from various farms was analyzed.

**Results:**

All isolates proved to be phenotypically Methicillin-susceptible *S. aureus* (MSSA), and none of them harbored *mec*A/C genes. The antimicrobial resistance against Tetracycline and Erythromycin were 15.62% and 3.12%, respectively. The isolates were assigned to ten sequence types in addition to five different clonal complexes using multilocus sequence typing (MLST). Sequence types ST133 (46.9%) and ST700 (21.9%) were the dominant types, and the majority of isolates were assigned to either CC133 (56.25%) or CC130 (34.4%). Twelve different *spa*-types were identified among isolates, while six isolates were not assigned to known *spa*-types. The dominant *spa*-type was t1773 (18.75%) which is known to be associated with CC130. All Tetracycline-resistant isolates harbored *tet* genes. The only Erythromycin-resistant isolate carried the *erm*(T) gene. The leucocidin genes *luk*F-PV (P83)/*lukM* were detected in 20 isolates (62.5%), while one isolate (ST522) carried a chimeric leukocidin.

**Conclusion:**

In conclusion, this study showed a considerable genetic diversity of *S. aureus* isolated from sheep and goat mastitis in Sardinia, Italy, and the prominent sensitivity to most of antimicrobial agents relevant for mastitis treatment. These findings inform about the local mastitis control strategies and highlight a low immediate public health risk from antimicrobial resistance in this setting.

## 1 Background

*Staphylococcus* (*S.*) *aureus* is incriminated in numerous human and animal diseases and represents a serious concern for both public and animal health. In humans, from a food-safety perspective, *S. aureus* is one of the predominant causes of food intoxication (Moawad et al., 2023), and is also considered as a frequent etiological agent of mastitis in dairy ruminants (Angelidis et al., 2020). Infectious mastitis outbreaks are primarily caused by bacterial infections, with *Staphylococcus* and *Streptococcus* species being the most commonly implicated pathogens. Among those, *S. aureus* stands out as a key etiological agent, often responsible for both clinical and subclinical forms of mastitis (Dore et al., 2016; Gelasakis et al., 2015; Marogna et al., 2010). The bacterium is frequently shed into the milk of infected animals (Li et al., 2017). In the rural Mediterranean regions, where small ruminant dairy farming is a cornerstone of agricultural economies, mastitis is particularly problematic. It is estimated that these areas contribute to two-thirds of global sheep milk production and a quarter of goat milk production, underscoring the importance of maintaining udder health in these animals (Dore et al., 2016). However, the economic burden of mastitis in these regions is substantial, with costs arising from veterinary treatments, animal culling, and the loss of milk production.

About 6.8 million sheep and 1 million goats in Italy provide milk for human consumption (Arif et al., 2023). Due to decreased milk quality and quantity, animal culling or replacement and increased medical treatments, mastitis – the most significant ovine and caprine disease –affects animal welfare and results in economic loss.

The annual incidence of clinical mastitis in small ruminants – sheep and goats – is generally lower than 5%. Nevertheless, in rare cases can exceed 30%–50%, causing mortality or culling of up to 70% of the flock (Mignacca et al., 2017). Despite the high prevalence, there is a lack of comprehensive studies detailing the local epidemiology, the population structure and genomic characteristics of mastitis-associated *S. aureus* in small ruminants, especially in Italy. Limited investigations have focused on bacterial prevalence in these animals, with notable studies such as those by Cannas et al. (2013) that primarily examined the situation in Sardinia.

Dairy goat grazing systems in Sardinia depend mainly on Sardinia’s traditional goat systems. These are favored because milk and cheese from grazing animals show higher nutritional and aromatic quality than those from stall-fed animals (Ruiz et al., 2009). Grazing behavior has important consequences as it affects the specific characteristics, features, and quality of animal dairy products (Claps et al., 2020). Consumption of raw milk and raw-milk cheeses is a rather common practice among farmers but also a “modern” trend among other consumers. Hence, in case of raw-milk consumption, the contamination of milk with *S. aureus* among other pathogens represents a public-health risk (Claeys et al., 2013).

Intramammary infection in animals caused by *S. aureus* strains is usually associated with a variety of virulence factors such as toxins, antigens and various resistance proteins (McMillan et al., 2016). Epidemiological research has shown that *S. aureus* is the most common cause of food poisoning in humans when pre-formed and temperature-stable staphylococcal enterotoxins (SEs) are ingested (Le Loir et al., 2003).

*Staphylococcus aureus* produces a wide range of toxins including numerous SEs and staphylococcal-enterotoxin-like- (SEl) toxins (Angelidis et al., 2020). Among SEs and SEls, the five classic SEs (*sea*, *seb*, *sec*, *sed* and *see*) have been reported to cause approximately 95% of Staphylococcal Food Poisoning (SFP) cases with *sea* being the most common cause (80%) and enterotoxins play a major role in the development of mastitis (Piccinini et al., 2012; Wang et al., 2017). Although there is a clear evidence that enterotoxins play a role in the inflammation and tissue damage noticed in mastitis, the overall scenario remains complex. The mere presence of enterotoxin genes does not consistently predict clinical outcomes. The ability of *S. aureus* to form biofilm is also considered a crucial virulence trait (Angelidis et al., 2020). Other virulence factors such as; host immune responses, and environmental conditions also significantly influence disease progression (Campos et al., 2022; Fang et al., 2019).

Genetic analyses have revealed the presence of biofilm-related genes (including *ica*A, *ica*D, *bap*, and *eno*) in numerous mastitis isolates, underscoring the potential biological importance of biofilm formation. Some *in vivo* experiments and studies on within-host bacterial evolution have associated biofilm development with persistent infections in the udder, indicating a possible role in the pathogenesis of bovine mastitis (Marbach et al., 2019).

However, most evidence for biofilm formation comes from *in vitro* studies. While these findings are compelling, direct *in vivo* evidence confirming the role of biofilms in mastitis pathogenesis remains scarce. The specific mechanisms involved and the extent to which biofilm formation alone influences clinical outcomes are still being explored, highlighting the need for further *in vivo* research to establish a definitive causal link (Marbach et al., 2019; Pedersen et al., 2021).

Data on antimicrobial resistance, as well as the clarification of the clonal diversity of *S. aureus* involved in clinical and subclinical mastitis in sheep and goats are crucial for identifying potential transmission pathways and for developing appropriate evidence-based control strategies for the affected herds.

Despite the global relevance of *S. aureus* in ruminant mastitis, comprehensive data on the molecular epidemiology, resistance, and virulence gene profiles of isolates from Sardinia are lacking. Such data are crucial both for animal-health management–through improved mastitis control–and for assessing food safety risks related to the consumption of raw milk and traditional cheeses.

Therefore, the aim of this study was to characterize *S. aureus* isolates from clinical mastitis cases in Sardinian sheep and goats, using whole-genome sequencing to assess their genetic diversity, antimicrobial resistance profiles and virulence determinants. The findings are expected to inform mastitis management strategies and contribute to the understanding of the zoonotic potential of *S. aureus* in this important dairy region.

## 2 Materials and methods

### 2.1 Isolation and phenotypic characterization of *Staphylococcus aureus*

**FIGURE 1:**
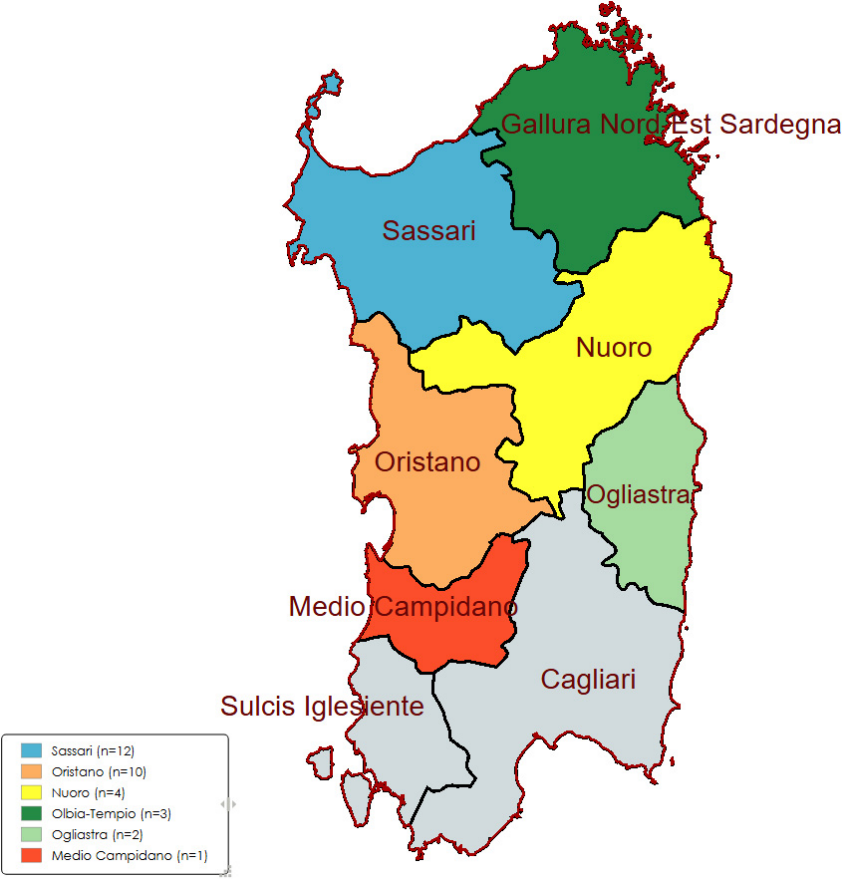
The sampling areas of 32 *S. aureus* isolates from clinical ovine and caprine mastitis in Sardinia, Italy.

A total of 32 *S. aureus* strains isolated from milk samples from 31 sheep and 1 goat infected during clinical mastitis outbreaks were collected between December 2021 and May 2022 from 25 different dairy herds in six provinces in Sardinia, Italy (Figure 1) by the National Reference Center for Sheep and Goat Mastitis. Farms were selected by convenience sampling through the National Reference Center for Sheep and Goat Mastitis, the Istituto Zooprofilattico Sperimentale della Sardegna (IZS), based on the submission of clinical mastitis cases. Consecutive clinical cases from each farm were included. No subclinical or environmental isolates were analyzed. Limited clinical data (species, age category, and clinical severity) were recorded when available.

The *S. aureus* strains were isolated from milk samples according to ISO 6888-1 under an ISO 17025-accredited quality management system as follows: Milk samples were thoroughly homogenized, and a serial dilution (e.g., 10^–1^ to 10^–4^) in sterile peptone water was prepared. From each dilution, 0.1 mL was inoculated onto Baird-Parker agar plates. That was followed by an aerobic incubation at 37 °C ± 1 °C for 24 ± 2 h. Isolates were confirmed by typical colony-shape: Black, shiny, convex colonies (1–1.5 mm diameter) with clear zones (halo) surrounded by an opaque zone. The biochemical tests including Coagulase and Catalase tests were applied, in addition to Gram stain.

The isolates distribution was as follows; [Sassari (*n* = 12), Oristano (*n* = 10), Nuoro (*n* = 4), Olbia-Tempio (*n* = 3), Ogliastra (*n* = 2) and Medio Campidano (*n* = 1) isolates] (Figure 1 and Supplementary Table 1).

Bacteriological cultivation was performed according to ISO 17025 of the National Mastitis Council Laboratory Handbook on Bovine Mastitis (National Mastitis Council [NMC], 2017). After confirmation biochemical and Gram stain of *S. aureus*, the isolates were further sent on nutrient transfer agar media to the Institute of Bacterial Infections and Zoonoses (IBIZ), Friedrich-Loeffler-Institut (FLI), Jena, Germany, for further investigation.

At FLI, swabs were inoculated in 10 mL of Mueller Hinton broth (Oxoid, Wesel, Germany) supplemented with 6.0% NaCl and incubated for 24 ± 2 h at 37 °C as a pre-enrichment broth.

After incubation, 50 μL of the broth was plated onto Baird-Parker Agar BPA (Oxoid, Wesel, Germany) in addition to Chromogenic medium for isolation and differentiation of MRSA (both CHROMagar Mastitis Pre-poured plates and Methicillin-containing CHROMagar MRSA pre-poured plates) (Mast Diagnostica GmbH, Reinfeld, Germany) at 37 °C for 24 h under aerobic conditions.

All isolates were confirmed as *S. aureus* using MALDI-TOF MS (Manukumar and Umesha, 2017; Yu et al., 2022). The interpretation of MALDI-TOF MS results was performed according to the manufacturer’s recommendations. The reference database was provided by Bruker (MBT-BDAL-8468). Multiplex real-time PCR assays were performed for genus and species identification (Velasco et al., 2014; Wang et al., 2014).

### 2.2 Phenotypic antimicrobial susceptibility testing

The pathogen’s susceptibility to an antimicrobial agent is measured through minimal inhibitory concentration (MIC) evaluation. The MICs for *S. aureus* isolates against different antimicrobial agents were performed using broth microdilution Micronaut Livestock-Equines plates (sifin diagnostics GmbH, Berlin, Germany) and VITEK-2 (AST-P592) (VITEK-2, bioMérieux Deutschland GmbH, Nürtingen, Germany). The isolates were tested for resistance to twenty-six different antimicrobial drugs assigned to different classes, Ampicillin, Cefquinom, Ciprofloxacin, Moxifloxacin, Ceftiofur, Doxycycline, Enrofloxacin, Florfenicol, Gentamicin, Neomycin, Linezolid, Teicoplanin, Trimethoprim/Sulfamethoxazole, Cefazolin, Cefoxitin, Clindamycin, Tigecycline, Fosfomycin, Fusidic acid, Nitrofurantoin, Oxacillin, Penicillin G, Rifampicin, Vancomycin, Erythromycin and Tetracycline. The detection of phenotypic Methicillin/Oxacillin resistance was additionally performed using *E*-test oxacillin (0.016–256 μg/mL) gradients strips (bioMérieux, Baden-Württemberg, Germany). Antimicrobial susceptibility interpretations followed CLSI M100 (2024) for agents primarily used in human medicine (e.g., Erythromycin, Cprofloxacin, Linezolid, Vancomycin) and CLSI VET08 (2018) for veterinary-specific antimicrobials (e.g., Ceftiofur, Enrofloxacin, Florfenicol, Tetracycline) (CLSI M100, 2024). For quality control of resistance testing, the reference strains *S. aureus* (DSM 2569) and *E. coli* (ATTCC^®^ 35218) were used (CLSI VET08, 2018).

### 2.3 DNA extraction and purification

A loopful from each fresh monoclonal culture was suspended in 1 ml sterilized phosphate buffer saline (PBS) and heat inactivated at 96 °C for 30 min. Additionally, cultures were preserved at −80 °C in cryotubes for further investigations.

Genomic DNA was extracted from bacterial cultures using QIAGEN Genomic-tip 20/G Kit (Qiagen GmbH, Hilden, Germany). The DNA extraction protocol was applied according to the instructions of the manufacturer with a prior in-house modification step, i.e., adding of a lysis mixture [10–20 μL lysostaphin, 20 μL lysozyme, 2 μL ribonuclease A (2 μL of 10 mg/mL) and 45 μL proteinase K] followed by an incubation at 37 °C for 2 h with slight shaking (Moawad et al., 2023).

The concentration and quality of eluted DNA was determined photometrically using a Colibri spectrophotometer (Titertek, Berthold Technologies GmbH & Co., KG, Bad Wildbad, Germany) and additionally measured using a Qubit 3 fluorometer (Fisher Scientific GmbH, Dreieich, Germany). The optical density ratio of purified DNA for all isolates ranged between 1.75 and 2.0 at 260/280 nm ratio with a concentration of ≥20 ng/μl. The prepared DNA was preserved at −20 °C for further investigations.

### 2.4 Determination of presence of Methicillin resistance genes

The Real-Time PCR was carried out in a 25-μL reaction mix including 10 μL of LC480 Probes Master PCR Reaction Mix (Roche Diagnostics, Mannheim, Germany), 3.25 μL of PCR-grade water (Roche Diagnostics, Mannheim, Germany), 1 μL of each primer, 0.25 μL of each probe 5 μL of template, and HPLC H_2_O for the no-template control. Amplification reactions were performed in a CFX96 Touch Real-Time PCR Detection Thermocycler System (Bio-Rad Laboratories GmbH, Feldkirchen, Germany). The assay detection limit was approximately 10^2^ CFU per reaction, consistent with previously validated protocols (Velasco et al., 2014).

PCR conditions were as follows; 1 × (50 °C, 2 min), 1 × (95 °C, 10 min) and 50 cycles (95 °C, 20 s; 60 °C, 40 s) (Velasco et al., 2014; Wang et al., 2014). *Staphylococcus aureus* (DSM 2569) was used as positive control in each reaction.

### 2.5 Whole genome sequencing (WGS)

The DNA of all isolates was sequenced using an Illumina MiSeq2000 platform (Illumina Inc., San Diego, CA) by paired-end sequencing producing 300 bp long reads. Sequencing libraries were created using the Nextera XT DNA Library Preparation Kit (Illumina Inc., San Diego, CA). Raw sequencing data were deposited in the European Nucleotide Archive (ENA) as BioProject PRJEB89476.

The bioinformatic analysis started with quality control of the raw paired-end reads. The Linux-based bioinformatics pipeline WGSBAC v. 2.2.01^1^ was used for data analysis. Unless other settings are mentioned, all tools were used in their default standard settings. For quality control of raw reads, FastQC v. 0.11.7 (Andrews, 2018) was used and the coverage was calculated. Based on raw reads, WGSBAC performed assembly using Shovill v. 1.0.4.^2^ Assembly quality statistics (N50, number of contigs, total genome size, GC content, and mean sequencing coverage per isolate) were summarized using QUAST v5.0.2. The quality of the assembled genomes was checked via QUAST v. 5.0.2 (Bankevich et al., 2012). In order to check for potential contamination on both reads and assemblies, the pipeline used Kraken 2 v. 1.1 (Wood et al., 2019) with the database Kraken2DB. For the detection of genetic markers for antimicrobial resistance and virulence, WGSBAC used the software ABRicate (v. 0.8.10)^3^ and the databases ResFinder (Florensa et al., 2022) NCBI (Sayers et al., 2020), and Virulence Factor Database (VFDB) (Chen et al., 2005). Moreover, WGSBAC used AMRFinderPlus (v. 3.6.10) (Feldgarden et al., 2019) which detects genes and point mutations leading to AMR.

AMRFinderPlus was used in organism-specific settings (i.e., *Staphylococcus aureus*). Platon was used for detection of plasmid borne contigs (Schwengers et al., 2020). In addition, the sequences on a previously published DNA-microarray (Monecke et al., 2011, 2013, 2016), were mapped against the probes.

For genotyping, WGSBAC used classical multilocus sequence typing (MLST) on assembled genomes using the software mlst v. 2.16.1 that incorporates the PubMLST database for the seven housekeeping genes of *S. aureus*.^4^ For high resolution genotyping, WGSBAC performed mapping-based genotyping using core genome single nucleotide polymorphisms (cgSNPs) identified by Snippy v. 4.3.0.^5^ The genome of the CC8 *S. aureus* NCTC 8325 (GenBank accession NC_007795.1) was used as reference. RAxML v. 8 (Stamatakis, 2014) was used for phylogenetic tree construction based on cgSNP alignment. The tree was rooted to the reference genome and visualized using the interactive Tree of Life (iTOL) v. 4 web tool. While the genome of *S. aureus* NCTC 8325 (ST8) was used as reference, we recognize that this strain is distantly related to some of our isolates (e.g., CC133, CC130). This may inflate SNP distances; therefore, results were interpreted qualitatively. In future analyses, lineage-specific references or cgMLST schemes will be considered.

In this study, *spa*-types were assigned and compared to known *spa*-types downloaded from the Ridom SpaServer^6^ (Mollerup et al., 2022). The *spa*-types was assigned using a free public application for searching and identification of known *spa* (Protein A) repeat types.^7^ This software provides results in the Kreiswirth and Ridom nomenclature (Supplementary Table 4).

## 3 Results

All 32 isolates were identified as *S. aureus* using MALDI-TOF MS and confirmed by real-time PCR (Supplementary Table 1). The isolates were distributed as follow; in Sassari (*n* = 12) isolates collected from 8 different farms, in Oristano (*n* = 10) from 8 different farms, in Nuoro (*n* = 4) from 4 different farms, in Olbia-Tempio (*n* = 3) from 2 different farms, in Ogliastra (*n* = 2) from 2 different farms and in Medio Campidano (*n* = 1) isolates (Table 1 and Supplementary Table 1).

**TABLE 1:** Sequence types (ST), clonal complexes (CC) and *spa* types of isolated *S. aureus*.

Strain ID	CC	ST	*Spa*-type	Municipality	Province
22CS0317	CC398	ST398	t1456	Seidlo	Oristano
22CS0318	CC130	ST700	t1773	Santa Giusta	Oristano
22CS0319	CC97	ST071	t524	Dorgali	Nuoro
22CS0321	CC130	ST700	t1773	Sassari	Sassari
22CS0322	CC130	ST700	t1773	Paulilatino	Oristano
22CS0323	CC133	ST133	N/A*	Noragugume	Nuoro
22CS0324	CC133	ST133	t5592	Mogoro	Oristano
22CS0325	CC133	ST133	N/A*	Borore	Nuoro
22CS0326	CC133	ST133	t2678	Ilbono	Ogliastra
22CS0327	CC522	ST522	N/A*	Arbus	Medio Campidano
22CS0328	CC133	ST133	t7008	Talana	Ogliastra
22CS0329	CC130	ST2011	t3568	Pattada	Sassari
22CS0330	CC130	ST2011	t3568	Pattada	Sassari
22CS0331	CC133	ST133	t3577	Oristano	Oristano
22CS0332	CC130	ST700	t1773	Simaxis	Oristano
22CS0333	CC133	ST133	t6058	Stintino	Sassari
22CS0334	CC130	ST700	t1773	Ozieri	Sassari
22CS0336	CC133	ST133	t1403	Oschiri	Olbia-Tempio
22CS0337	CC133	ST133	t1403	Oschiri	Olbia-Tempio
22CS0338	CC133	ST132	t6056	Nule	Sassari
22CS0339	CC133	ST133	t6056	Seidlo	Oristano
22CS0340	CC133	ST133slv	t6056	Irgoli	Nuoro
22CS0341	CC130	ST521	t524	Ozieri	Sassari
22CS0342	CC130	ST700	N/A*	Palmas Arborea	Oristano
22CS0343	CC133	ST133	t2678	Gonnosnò	Oristano
22CS0344	CC130	ST700	t1773	Borutta	Sassari
22CS0345	CC133	ST133slv	t6056	Buddusò	Olbia-Tempio
22CS0346	CC130	ST130slv	N/A*	Palmas Arborea	Oristano
22CS0347	CC133	ST133	t3585	Bono	Sassari
22CS0348	CC133	ST133	t2678	Sassari	Sassari
22CS0349	CC133	ST133	N/A*	Sassari	Sassari
22CS0350	CC133	ST133	t2678	Ittireddu	Sassari

*N/A, not applicable.

Among the isolates, no Methicillin resistance was revealed, and all isolates were confirmed as Methicillin sensitive *S. aureus* (MSSA) by absence of growing on chromogenic MRSA agar media and lack of amplification of the *mec*A gene by real-time PCR (Supplementary Tables 2, 3).

### 3.1 Phenotypic antimicrobial resistance profiles

All *S. aureus* were phenotypically sensitive to Ampicillin, Cefquinom, Ciprofloxacin, Moxifloxacin, Ceftiofur, Doxycycline, Enrofloxacin, Florfenicol, Gentamicin, Neomycin, Linezolid, Teicoplanin, Trimethoprim/Sulfamethoxazole, Cefazolin, Cefoxitin, Clindamycin, Tigecycline, Fosfomycin, Fusidic acid, Nitrofurantoin, Oxacillin, Penicillin G, Rifampicin and Vancomycin. Only few isolates showed resistance against Erythromycin (*n* = 1; 3.12%) and Tetracycline (*n* = 5; 15.62%). No isolates were identified as multi-drug resistant (MDR) or MRSA (Supplementary Table 2).

### 3.2 Genetic characterization of *Staphylococcus aureus*

**FIGURE 2:**
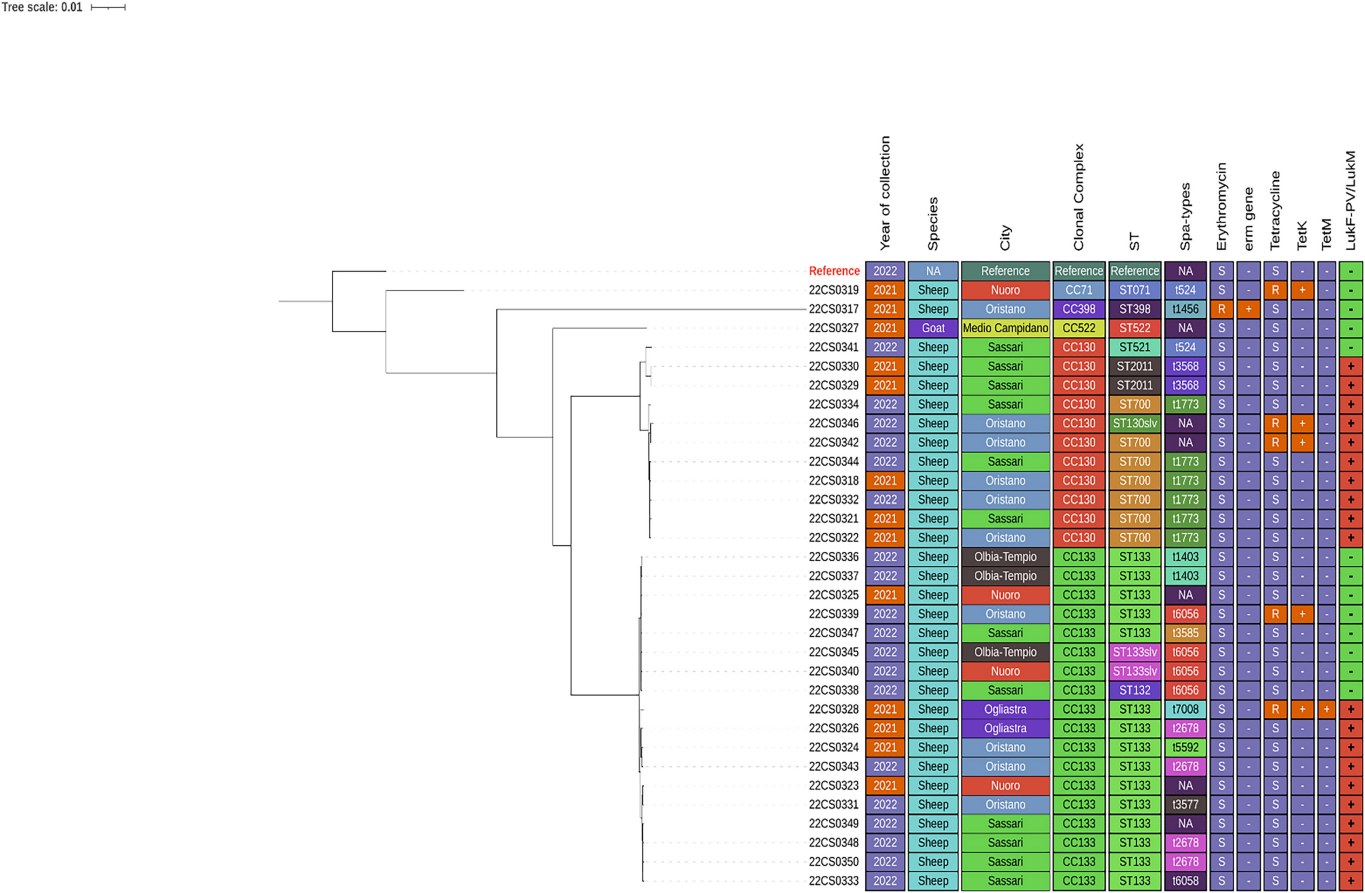
Maximum likelihood tree of 32 *S. aureus* isolated from clinical mastitis cases from 25 farms in Sardinia using WGS analysis.

All 32 *S. aureus* isolates were sequenced and the coverage and genomic size of different isolates were showed in Supplementary file (Supplementary Tables 3, 4). Ten different MLST sequence types (ST) were identified. The clonal complex (CC522) was identified in the single goat isolate from Medio Campidano (ST522) in 2021 (Figure 2 and Table 1). CC130 was identified among 11 sheep-isolates from 8 different farms located in Oristano and Sassari [ST700 (7), ST2011 (2), ST521 (1) and ST130slv (1)] between 2021 and 2022. In addition, CC133 was identified among 18 sheep-isolates from 16 different farms located in Nuoro, Oristano, Ogliastra, Olbia-Tempio and Sassari [ST133 (15), ST132 (1) and ST133slv (2)] between 2021 and 2022. CC398 was identified in one sheep-isolate from Seidlo (ST398) in 2021. Lastly, CC97 was identified in one sheep-isolate from Nuoro (ST071) in 2021 (Table 1 and Supplementary Table 1).

The most frequently confirmed CC was CC133 to which 18 isolates were assigned. A majority (*n* = 15) of them were assigned to ST133, except for one isolate of ST132 and two ST133 single locus variants.

Eleven isolates belonged to CC130 and were approved as seven isolates of ST700 (21.9%), two isolates of ST2011 and one isolate each of ST130 (slv) and ST522. Of note, ST398, clonal complex (CC398), as frequently observed in livestock was identified in only one isolate. Twelve different *spa*-types were assigned for most of the isolates with few exceptions not identified *spa*-types (NA) because the repeat region was split across contigs, precluding confident assignment. These were denoted as “N/A” (Figure 2 and Table 1).

### 3.3 Phenotypic - genotypic correlation of antimicrobial resistance

The isolates showed 100% compatibility in phenotypic and genotypic resistance profiles for all tested drugs (Supplementary Tables 2–4).

Neither *mec*A nor *mecC* were detected which was fully in accordance to the phenotypic test results. The Erythromycin resistance gene *erm*(T) was identified in one isolate (3.12%) of isolates. That was the only isolate belonging to ST398, which also was phenotypically Erythromycin resistant.

The presence of the Tetracycline-resistance gene *tet*(K), was identified in five isolates that were all phenotypically resistant to Tetracycline and were assigned to ST71 (“CC97”) and CC133 (2 isolates each) or CC130 (2 isolates). Additionally, one Tetracycline-resistance CC133 isolate was positive for *tet*(M) gene (Supplementary Tables 3, 4).

Platon analysis indicated that most AMR and virulence genes were located on chromosomal contigs (91%), with a minority (9%) associated with putative plasmid contigs.

### 3.4 Virulence associated factors

Seventy-one virulence genes were identified in 32 *S. aureus* isolates using WGS and analysis by Virulence Factor Database (VFDB) (Chen et al., 2005) (Supplementary Tables 3, 4). They were assigned to five functional categories [adherence related virulence factors (*n* = 14), toxins; including toxin system genes (*n* = 15), host immune evasion genes (*n* = 20), exoenzymes (*n* = 10) and iron acquisition- and metabolism-related genes (*n* = 7)].

The leukocidin genes *luk*F-PV (P83)/*luk*M were detected in 20 isolates (62.5%) that belonged to either CC130 or CC133.

One isolate (ST522) carried a chimeric leukocidin which is the same as in GenBank CP138360; (Monecke et al., 2025) where the sequence of the *luk*S- component is identical to *luk*P from equine strains of *S. aureus* while the *luk*F component is a chimera comprising parts of equine-associated *luk*Q and of *luk*F-PV (P83). The results of alignment analysis are provided in Supplementary Table 4.

Eleven isolates (four belonging to CC130, six to CC133 and the one ST522) harbored the toxic shock syndrome toxin gene (*tst*1) and enterotoxin genes *sec* and *sel*. In one CC133 isolate, *sel* alone was identified.

No other enterotoxin genes were found. None of the enterotoxin gene cluster genes was detected among the isolates. Genes *chp* and *scn* (encoding chemotaxis-inhibiting protein CHIPS and a staphylococcal complement inhibitor) were identified in the single ST398 isolate.

Genes encoding epidermal cell differentiation inhibitors *edinB* and exfoliative toxin *etD2* were found in all CC130 sequences except for one that apparently lacked *edinB*. Other genes encoding epidermal cell differentiation inhibitors or exfoliative toxins were not detected.

The tree was rooted to the reference genome (CC8/ST8) and visualized using the interactive Tree of Life (iTOL) v. 4 web tool. Data included are; Survey year, Province, species, clonal complexes, sequence types, *spa*-types, Erythromycin resistance, *erm* gene carriage, Tetracycline resistance, *tet*(K&M) and *luk*F-PV (P83)/*luk*M (Figure 2).

## 4 Discussion

The present study provides insights into the genetic characteristics, antimicrobial resistance (AMR) profiles and virulence associated factors of *Staphylococcus aureus* isolates from clinical mastitis cases in small ruminants in Sardinia, Italy. Our findings underscore the significant role of *S. aureus* as a prevalent pathogen in mastitis within this region, aligning with broader understanding of its impact on dairy sheep and goats globally (Angelidis et al., 2020). The study’s focus on whole-genome sequencing (WGS) offering a high-resolution view of the *S. aureus* population structure and contributing to the limited comprehensive data available for Italy in sheep and goats, particularly Sardinia.

One of the most prominent findings is the dominance of ST133 (46.9%) and ST700 (21.9%) among the identified sequence types, with the majority of isolates assigned to CC133 (56.25%) or CC130 (34.4%). The prevalence of ST133 in our study aligns with previous reports from Mediterranean flocks, suggesting a regional endemicity or widespread dissemination of this lineage in small ruminant mastitis (Dore et al., 2016). For instance, a study in Greece also identified ST133 as a prevalent lineage in ovine mastitis (Gelasakis et al., 2015). In contrast, other regions, particularly in Northern Europe, often reported ST398 as the predominant livestock-associated *S. aureus* lineage (Moawad et al., 2023; Monecke et al., 2011; Li et al., 2022). The results of this study identified ST398 in only one isolate, highlighting the regional differences in *S. aureus* clonal epidemiology. The high prevalence of ST700, belonging to CC130, is also noteworthy and has been previously suggested as a prevalent *S. aureus* lineage associated with ovine mastitis in Sardinia (Angelidis et al., 2020). This consistency reinforces the importance of these clonal complexes in the local epidemiology of *S. aureus* mastitis.

In this study, all isolates were phenotypic Methicillin-susceptible *S. aureus* (MSSA), with no detection of *mec*A/C genes, is a reassuring outcome given the global concern over Methicillin-resistant *S. aureus* (MRSA) in both livestock and humans. This contrasts with some reports from other parts of Italy and Europe where MRSA, including LA-MRSA ST398, has been detected in dairy animals, at varying prevalence (Moawad et al., 2023; Monecke et al., 2013, 2016). This absence of MRSA in our cohort suggests a low immediate public health risk from Methicillin resistance in this specific setting, which is crucial for mastitis control strategies and food safety in a region known for raw milk and traditional cheese production. It is relevant to mention that, as livestock associated-CC398 usually refers to MRSA and the single identified ST398 isolate in this study was a MSSA, it is assumed to be from a “human” background.

The antimicrobial susceptibility profiles observed in this study reveal a generally high sensitivity of *S. aureus* isolates to most antimicrobial agents relevant for mastitis treatment.

This is a positive indicator for effective therapeutic interventions in Sardinian small ruminant farms. Specifically, the low rates of resistance to Erythromycin (3.12%) and Tetracycline (15.62%) are encouraging. The phenotypic and genotypic resistance profiles showed 100% concordance, reinforcing the reliability of our findings regarding AMR.

The detection of the Erythromycin resistance associated gene *erm*(T) in the single Erythromycin-resistant isolate (ST398) and the Tetracycline resistance genes *tet*(K) and *tet*(M) in Tetracycline-resistant isolates provides a clear genetic basis for the observed resistance. The presence of *tet*(K) in isolates from different clonal complexes (ST71, CC133, CC130) suggests that these resistance genes are horizontally transferable or widely disseminated within the *S. aureus* population in this region. The additional presence of *tet*(M) in one CC133 isolate enhances the potential for co-occurrence of different resistance mechanisms.

While the overall resistance rates are low, the presence of even limited resistance to commonly used antibiotics like Tetracycline and Erythromycin warrants continuous surveillance. Tetracyclines are broad-spectrum antibiotics frequently used in veterinary medicine, and their continued use can exert selective pressure, potentially leading to an increase in resistance (Caneschi et al., 2023).

The whole-genome sequencing analysis revealed significant genetic diversity among the *S. aureus* isolates, with ten different sequence types identified. The dominance of ST133 and ST700, along with the presence of other STs and clonal complexes (CC133, CC130, CC97, CC398 and CC522), indicates a diverse *S. aureus* population circulating in Sardinian small ruminant farms.

The identification of numerous virulence-associated genes underscores the pathogenic potential of these *S. aureus* isolates in causing mastitis. Seventy-one virulence genes were identified, categorized into adherence, toxins, host immune evasion, exoenzymes and iron acquisition/metabolism. Notably, the leukocidin genes *luk*F-PV (P83)/*luk*M were detected in 62.5% of the isolates. These genes encode a leukocidin specific to ruminants and are distinct from the classic Panton-Valentine leukocidin (PVL)–*luk*F-PV/*luk*S-PV–commonly associated with human *S. aureus* strains (Monecke et al., 2025). High *luk*M carriage aligns with previous reports linking this leukocidin to severe clinical presentation and persistence in small ruminant mastitis (Fang et al., 2019). Its role in tissue necrosis and neutrophil lysis supports its significance as a key virulence determinant in ovine infections.

This distinction is crucial to prevent misinterpretation and to accurately reflect the host specificity and zoonotic potential of these strains. The presence of *luk*F-PV (P83)/*luk*M has been linked to enhanced virulence in animal infections, particularly in the pathogenesis of mastitis (Moawad et al., 2023). Additionally, the detection of various virulence factors, including this ruminant-specific leukocidin, highlights the pathogenic capacity of these *S. aureus* strains in animals. Although this leukocidin is not directly implicated in human foodborne illness, the presence of virulent *S. aureus* in milk remains a concern due to its potential to cause udder infections, thereby affecting both the quality and yield of milk (Moawad et al., 2023).

The chimeric leukocidin carried by the ST522 isolate, with components similar to equine strains, highlights the complex genetic recombination and adaptation of *S. aureus* to different hosts and environments (Monecke et al., 2025).

*Staphylococcuy aureus* is a known cause of food intoxication due to the production of heat-stable enterotoxins, and even in the absence of the most common types, vigilance is necessary (Moawad et al., 2023). Furthermore, the detection of the toxic shock syndrome toxin gene (*tst*1) and enterotoxin genes *sec* and *sel* in some is significant. While the absence of other classic enterotoxin genes (e.g., *sea, seb, sed, see*) suggests that these isolates might not be primarily associated with foodborne illness (Chen et al., 2023), the presence of *sec* and *sel* enterotoxin genes in some isolates in the current study cannot be overlooked. Although these are less frequently implicated in severe food poisoning outbreaks compared to classic enterotoxins, their presence in milk from infected animals poses a potential food-safety concern, particularly in raw-milk cheese production common in Sardinia. These findings underscore the need for stringent hygiene and heat-treatment control in traditional dairy processing.” Identification of genes like *chp* and *scn* in the ST398 isolate, known to be co-localized on hemolysin-beta-converting phages, indicates cross infection between farmers and livestock (Howden et al., 2023).

Although the absence of MRSA suggests a low antimicrobial resistance burden, toxin-mediated risks, especially from enterotoxin genes (*sec, sel, tst1*), remain relevant for food safety. Therefore, our statement regarding “low public risk” refers specifically to antimicrobial resistance, not toxin hazards. The presence of other antimicrobial resistance genes, such as *erm*(T) and *tet*(K)/*tet*(M) highlights the broader issue of antimicrobial resistance in the food chain (Pennone et al., 2022).

While these resistances are currently at low levels, their potential for dissemination through the consumption of contaminated milk or dairy products, or direct contact with animals, remains a concern. The epidemiological significance of these resistance genes, and their possible routes of acquisition and spread (e.g., through horizontal gene transfer or selective pressure from antibiotic use in veterinary medicine), needs to be continuously monitored and updated.

BLAST comparison showed >99.5% identity of the ST398 isolate with human-derived MSSA genomes (GenBank CP018746, CP017091), supporting its likely human origin rather than livestock-associated lineage.

Comparison with publicly available small-ruminant *S. aureus* genomes (*n* = 120; NCBI, 2024) revealed that ST133 and ST700 are among the most common global lineages in sheep mastitis, whereas CC398 and CC522 are less frequent. This suggests a regional predominance of CC133/CC130 lineages in Mediterranean herds.

The practice of consuming raw milk or raw-milk cheeses, while culturally significant and offering certain nutritional benefits, carries an inherent risk of exposure to various pathogens, including *S. aureus*. Therefore, robust hygiene protocols at the farm level, proper handling and processing of milk, and consumer awareness regarding the risks associated with raw milk consumption are crucial to mitigate potential public health hazards. The significance of our findings for food safety and zoonotic risk lies in informing these preventative measures and guiding public health policies.

### 4.1 Study limitations

While this study provides an insight into the *S. aureus* population in Sardinian small ruminant mastitis, it is important to acknowledge certain limitations. Firstly, the sample size of 32 *S. aureus* isolates, collected from 25 dairy herds in six provinces in Sardinia, while representative of the sampled farms, may not fully capture the entire genetic diversity and prevalence of *S. aureus* across all small ruminant farms in Sardinia or other regions of Italy. Secondly, the study focused exclusively on *S. aureus* isolates from clinical mastitis cases. This approach may not provide a complete picture of the *S. aureus* population, as subclinical mastitis, which is often more prevalent and can act as a reservoir for infection, was not directly investigated in terms of *S. aureus* isolation and characterization.

Finally, the study’s cross-sectional design provides a snapshot of the *S. aureus* population at a specific time (December 2021 to May 2022). Longitudinal studies would be beneficial to understand the dynamics of *S. aureus* clonal lineages, the emergence and spread of antimicrobial resistance, and the long-term impact of control strategies over time.

## 5 Conclusion

This study contributes to the understanding of *Staphylococcus aureus*-induced mastitis in small ruminants in Sardinia, Italy. By employing whole-genome sequencing, we have characterized the genetic diversity, antimicrobial resistance profiles, and virulence determinants of *S. aureus* isolates. Our findings confirm the prominent role of *S. aureus* in mastitis in this region identified as dominant lineages. The reassuring absence of MRSA, coupled with low resistance rates to other antimicrobials, highlights a favorable current situation regarding antimicrobial resistance in this specific setting. However, the presence of specific resistance genes and a diverse array of virulence factors underscores the continuous need for vigilance.

The findings of this study show that various clonal complexes have the potential to spread over different geographical areas (e.g., CC130 and CC133). Additionally, a significant number of different sequence types was found and geographically widely distributed among dairy farms.

The insights gained from this research are crucial for informing local mastitis control strategies, promoting prudent antibiotic use, and safeguarding both public and animal health, particularly in the context of raw milk production. While the immediate public health risk from antimicrobial resistance appears low, ongoing surveillance and adherence to robust hygiene practices are essential to prevent the emergence and spread of multidrug-resistant strains. Future research should build upon these findings through longitudinal studies, investigations into subclinical mastitis, and functional characterization of virulence factors to develop more effective and sustainable control measures against *S. aureus* mastitis in small ruminants. It should be noted that this investigation was based on a relatively small, cross-sectional sample collected over a limited period. Therefore, the conclusions apply specifically to the sampled herds and period and should not be generalized to all Sardinian or Mediterranean small-ruminant populations.

## Data Availability

The datasets presented in this study can be found in online repositories. The names of the repository/repositories and accession number(s) can be found in this article/Supplementary material.

## Footnotes

1
https://gitlab.com/FLI_Bioinfo/WGSBAC

2
https://github.com/tseemann/shovill

3
https://github.com/tseemann/abricate

4
https://pubmlst.org/organisms/staphylococcus-aureus

5
https://github.com/tseemann/snippy

6
https://spa.ridom.de/spatypes.shtml

7
https://spatyper.fortinbras.us/
